# Molecular analyses and phylogeny of the herpes simplex virus 2 US9 and glycoproteins gE/gI obtained from infected subjects during the Herpevac Trial for Women

**DOI:** 10.1371/journal.pone.0212877

**Published:** 2019-03-08

**Authors:** Kelsey L. Rowe, Miguel A. Minaya, Robert B. Belshe, Lynda A. Morrison

**Affiliations:** 1 Department of Molecular Microbiology and Immunology, Saint Louis University School of Medicine, St. Louis, Missouri, United States of America; 2 Department of Internal Medicine, Division of Infectious Diseases, Saint Louis University School of Medicine, St. Louis, Missouri, United States of America; University of Pittsburgh School of Medicine, UNITED STATES

## Abstract

Herpes simplex virus 2 (HSV-2) is a large double-stranded DNA virus that causes genital sores when spread by sexual contact and is a principal cause of viral encephalitis in newborns and infants. Viral glycoproteins enable virion entry into and spread between cells, making glycoproteins a prime target for vaccine development. A truncated glycoprotein D2 (gD2) vaccine candidate, recently tested in the phase 3 Herpevac Trial for Women, did not prevent HSV-2 infection in initially seronegative women. Some women who became infected experienced multiple recurrences during the trial. The HSV U_S_7, U_S_8, and U_S_9 genes encode glycoprotein I (gI), glycoprotein E (gE), and the US9 type II membrane protein, respectively. These proteins participate in viral spread across cell junctions and facilitate anterograde transport of virion components in neurons, prompting us to investigate whether sequence variants in these genes could be associated with frequent recurrence. The nucleotide sequences and *d*N/*d*S ratios of the *U*_*S*_*7-U*_*S*_*9* region from viral isolates of individuals who experienced multiple recurrences were compared with those who had had a single episode of disease. No consistent polymorphism(s) distinguished the recurrent isolates. In frequently recurring isolates, the *d*N/*d*S ratio of *U*_*S*_*7* was low while greater variation (higher *d*N/*d*S ratio) occurred in *U*_*S*_*8*, suggesting conserved function of the former during reactivation. Phylogenetic reconstruction of the *U*_*S*_*7-U*_*S*_*9* region revealed eight strongly supported clusters within the 55 U.S. HSV-2 strains sampled, which were preserved in a second global phylogeny. Thus, although we have demonstrated evolutionary diversity in the *U*_*S*_*7-U*_*S*_*9* complex, we found no molecular evidence of sequence variation in *U*_*S*_*7-U*_*S*_*9* that distinguishes isolates from subjects with frequently recurrent episodes of disease.

## Introduction

Herpes simplex virus 2 (HSV-2) is a large, enveloped dsDNA virus with an icosahedral capsid, a dense layer of tegument proteins, and a host cell-derived lipid envelope studded with viral glycoproteins [1]. HSV-2 traditionally causes genital sores when spread by sexual contact, although due to changing sexual practices HSV-1 causes an equivalent proportion of HSV genital infections [2–4]. Because subclinical infections are common, many individuals are unaware that they are infected. Seroprevalence of HSV-2 in 14 to 49-year-olds in the United States between 2005 and 2010 was estimated to be 15.7% [5]. Herpes simplex encephalitis, caused by HSV-2 or HSV-1, is the most common cause of viral encephalitis in infants [6]. Viral shedding of HSV from the maternal genital tract can result in transmission of the virus from mother to child, often leading to encephalitis and multi-organ disseminated disease [4, 7, 8].

During initial infection, HSV initiates cytolytic replication in epithelial cells. Intranuclear inclusions form in infected cells and the infection induces cell-cell fusion to produce multinucleated giant cells. Viral replication causes cell lysis, leading to detachment of the outer epidermis and formation of blisters. HSV then penetrates the dermis and peripheral sensory nerve termini. Retrograde transport of virus through axons of sensory neurons brings virus to the ganglia where it establishes latent infection. From latency the virus can reactivate, at which time virion components are transported anterogradely through the axon and assemble into virus particles at the peripheral nerve ending. Virus crosses from there to epithelial cells where it replicates to cause recurrent lesions and can reestablish latency. Thus, symptomatic episodes can recur throughout an infected individual’s lifetime [9].

The U_S_7, U_S_8, and U_S_9 genes encode glycoprotein I (gI), glycoprotein E (gE), and US9 type II membrane protein, respectively [10–14]. An additional open reading frame in HSV-1, *U*_*S*_*8A*, overlaps the 3’ portion of the U_S_8 gene and the region between *U*_*S*_*8* and *U*_*S*_*9* [15]. During recurrent disease episodes, gE and gI form a protein complex (gE/gI) which functions with US9 to facilitate anterograde viral spread through neurons [16, 17], but the mechanism of enhanced transport is not fully understood. One hypothesis proposes gE/gI and US9 promote the accumulation of membrane vesicles containing virion components and enveloped virions in the trans-Golgi network (TGN) of neuronal cell bodies. Virion components in the TGN-like membranes are then likely loaded onto kinesin motors. This makes the role of gE/gI and US9 vital for axonal transport of the virus. [16]. A similar theory, the “missorting model,” proposes that gE/gI and US9 share TGN sorting motifs, with loss of gE/gI or US9 leading to misrouted capsids and enveloped virions that cannot enter axons to be transported [11]. The HSV-1 US8A protein also facilitates neuronal spread of virus particles into the central nervous system (CNS) from peripheral sites [18] but the precise mechanism promoting neuroinvasion is not known. In addition to its role in neuronal transport, the gE/gI complex acts as an Fc receptor (FcRγ). This FcRγ inhibits antiviral IgG activity by attaching to the Fc portion of an antibody molecule specifically bound via its Fab domain to a viral antigen on the virion surface. In this way, antibody bipolar bridging blocks all activities mediated by the IgG, allowing viral evasion [19].

HSV glycoprotein D (gD) mediates specific entry into cells, and has therefore been considered a prime target for vaccine development [20]. However, a truncated glycoprotein D2 (gD2) vaccine candidate recently tested in the phase 3 Herpevac Trial for Women was not successful in preventing HSV-2 infection or disease [21]. Some women who became infected during the trial experienced multiple episodes of recurrent disease, while others did not. To explore this phenotypic variation, we investigated whether amino acid sequence differences in the gE/gI and US9 proteins are associated with more frequent recurrence. Sequences of the corresponding U_S_7, U_S_8 and U_S_9 genes of HSV-2 isolates obtained during the Herpevac Trial from subjects who had a single episode of disease were compared with those who had experienced three or more recurrent disease episodes. We also evaluated selection pressures on these viral genes and the phylogenetic associations among the HSV-2 U_S_7-U_S_9 genes sequenced from the U.S. and from diverse parts of the world.

## Materials and methods

### Study design and sample collection

In the Herpevac Trial for Women, subjects were vaccinated at months 0, 1, and 6 with either the investigational vaccine, consisting of 20 μg of glycoprotein D2 from HSV-2 strain G in alum and 3-O-deacylated monophosphoryl lipid A as an adjuvant, or a control hepatitis A vaccine. The non-recurrent endpoint was prevention of genital herpes disease caused by HSV-1, HSV-2, or both from month 2 (1 month after dose 2) through month 20 [21, 22]. Swab samples were collected from subjects experiencing either “non-recurrent” or “recurrent” symptomatic disease. Here we define “non-recurrent” isolates as those derived from subjects who experienced a single disease episode during the trial. The “recurrent” isolates were taken from the last disease episode of subjects who had 3 or more recurrences during the trial. A total of 12 non-recurrent and 8 recurrent isolates were obtained from 20 subjects who became infected with HSV-2 during the vaccine trial. The study was approved by the Saint Louis University Institutional Review Board (IRB number 27007). Subjects provided written consent to future use of their samples.

### Cells and viruses

Vero cells (African green monkey kidney epithelial origin), were maintained in Dulbecco’s modified Eagle's medium (DMEM) supplemented with 3% newborn calf and 3% bovine growth sera, 100 IU/ml penicillin and 0.1 mg/ml streptomycin (1x P/S), and were originally obtained from David Knipe. HSV-2 swab isolates in transport medium collected during the trial were thawed and 100 μl were inoculated onto Vero cell monolayers in 75cm^2^ flasks. Monolayers were incubated until cytopathic effect reached 100%, and cell lysate stocks of the isolates were prepared [23].

### DNA isolation and sequencing

Viral DNA was purified from a portion of each virus lysate using QIAmp DNA Mini kit (Qiagen, Valencia, CA) according to the manufacturer’s recommendations. *U*_*S*_*7*, *U*_*S*_*8*, *U*_*S*_*8A* and *U*_*S*_*9* were PCR-amplified using strain-specific overlapping primers (Table 1). Amplification reactions used a reaction mixture containing 0.75 μl of forward and reverse primer (10 mM), 2.5 μl 10X AccuPrime^TM^ *Pfx* Reaction Mix (Invitrogen), 2.5 μl Betaine solution (5M) (Sigma), 2 μl MgCl_2_ (50mM), 1.5 μl DMSO, 0.5 μl *Taq* DNA polymerase, 3 μl of template DNA (20–30 ng/μl) in a total reaction vol. of 25 μl. The amplification parameters consisted of an initial denaturing step of 2 min at 95°C, followed by 39 cycles of 20 sec denaturing at 95°C, 30 sec annealing at primer-specific temperature of 56°C, and 3 min extension at 68°C, followed by a final extension step of 5 min at 68°C. Sanger sequencing of purified PCR products was conducted by GeneWiz, Inc. (South Plainfield, NJ).

**Table 1:** Primers used for amplification of HSV-2 *U*_*S*_*7*, *U*_*S*_*8* and *U*_*S*_*9*.

Gene	Name	Genome location^a^	Sequence 5’ to 3’
**HSV-2 Us7**	Fwd1_US7_HSV2	142079	TCA TCG GCG GTA TTG CGT T
	Rev1_US7_HSV2	142906	GCC GTT ATA CAC AAA CGT CCC
**HSV-2 Us7**	Fwd2_US7_HSV2	142708	GCG TTC ACC TTG TGT CGC TC
	Rev2_US7_HSV2	143659	CCC ATC CAT TAC CCG CCT C
**HSV-2 Us8-Us9**	Fwd1_US8-9_HSV2	143608	TGC GTG TGT TGG ATC CCT TT
	Rev1_US8-9_HSV2	144457	GGG ACA CCT CGG GGA TAA CA
**HSV-2 Us8-Us9**	Fwd2_US8-9_HSV2	143876	GTT TGG GTC GTA TCG TGC CT
	Rev2_US8-9_HSV2	144661	GCT GCG GGT GAT ACA GAC AA
**HSV-2 Us8-Us9**	Fwd3_US8-9_HSV2	144604	CGT CTG GAT GCG GTT TGA C
	Rev3_US8-9_HSV2	145459	TTA CCA GAG GAC GGA CGG A
**HSV-2 Us8-Us9**	Fwd4_US8-9_HSV2	145377	ATA GCG AGG GGC GTA AAT CTC
	Rev4_US8-9_HSV2	146118	ATT TAG CGG AGC AGC CAC AC

^a^ Nucleotide position within the HSV-2 strain HG52 (JN561323) complete genome.

### Sequence analyses

Chromatograms of each sequence were visually inspected for overlapping peaks that could indicate a single nucleotide variation (SNV) comprising a portion of the viral population, but none was found. Complementary strands were assembled and verified using the program Sequencher 4.2.2 (Gene Codes Corporation, Ann Arbor, Michigan, U.S.A). The obtained sequences were aligned and adjusted manually using *MEGA* (v7.0.14) [24]. Improvement of the nucleotide alignments was performed using amino acid translations and by comparing our sequences with the previously published cDNA from HSV-2 strain HG52 (JN561323), a laboratory HSV-2 strain from Scotland [25], and SD90e (KF781518), a clinical isolate of HSV-2 from South Africa [26] (Table 2). These comparisons were also used to check the genic-intergenic boundaries in the *U*_*S*_*7-U*_*S*_*8-U*_*S*_*8A*-*U*_*S*_*9* complex. These alignments were then used to construct amino acid sequence models of the gE, gI and US9 proteins, and the putative HSV-2 homolog of HSV-1 US8A for each strain sampled so that the SNVs and insertions/deletions (INDELs) observed in different sequences could be easily compared.

**Table 2:** Genomes and accession numbers.

Virus	Strain	Host	Anatomical region	Isolate history	Collection year	Country of origin	GenBank accession no.
HSV-2	Sample 06	Human	Genital	Non-recurrent	2006	United States	MH612582
HSV-2	Sample 10	Human	Genital	Non-recurrent	2005	United States	MH612583
HSV-2	Sample 14	Human	Genital	Recurrent	2004	United States	MF564035
HSV-2	Sample 15	Human	Genital	Non-recurrent	2004	United States	MH612584
HSV-2	Sample 17	Human	Genital	Non-recurrent	2007	United States	MH612585
HSV-2	Sample 18	Human	Genital	Non-recurrent	2006	United States	MH612586
HSV-2	Sample 19	Human	Genital	Recurrent	2008	United States	MF564037
HSV-2	Subject 02	Human	Genital	Non-recurrent	2004	United States	MH612587
HSV-2	Subject 09	Human	Genital	Recurrent	2008	United States	MH612588
HSV-2	Subject 16	Human	Genital	Non-recurrent	2008	United States	MH612589
HSV-2	Subject 18	Human	Genital	Recurrent	2004	United States	MH612590
HSV-2	Subject 23	Human	Genital	Non-recurrent	2008	United States	MH612591
HSV-2	Subject 27	Human	Genital	Non-recurrent	2005	United States	MH612592
HSV-2	Subject 32	Human	Genital	Recurrent	2005	United States	MH612593
HSV-2	Subject 35	Human	Genital	Recurrent	2006	United States	MH612594
HSV-2	Subject 39	Human	Genital	Non-recurrent	2006	United States	MH612595
HSV-2	Subject 40	Human	Genital	Recurrent	2008	United States	MH612596
HSV-2	Subject 42	Human	Genital	Non-recurrent	2005	United States	MH612597
HSV-2	Subject 46	Human	Genital	Recurrent	2008	United States	MH612598
HSV-2	Subject 53	Human	Genital	Non-recurrent	2008	United States	MH612599
HSV-2	2003 16029	Human	Genital	Clinical	2003	United States	KX574903
HSV-2	2004 20601	Human	Genital	Clinical	2004	United States	KX574904
HSV-2	2006 38659	Human	Genital	Clinical	2006	United States	KX574907
HSV-2	2006 49895	Human	Genital	Clinical	1994	United States	KX574868
HSV-2	2006 50074	Human	Genital	Clinical	1995	United States	KX574869
HSV-2	2007 22031	Human	Genital	Clinical	2007	United States	KX574870
HSV-2	2007 40807	Human	Genital	Clinical	2007	United States	KX574882
HSV-2	2008 7539	Human	Genital	Clinical	2008	United States	KX574887
HSV-2	2008 16404	Human	Genital	Clinical	2008	United States	KX574884
HSV-2	2008 37744	Human	Genital	Clinical	2008	United States	KX574886
HSV-2	2009 1245	Human	Genital	Clinical	1994	United States	KX574888
HSV-2	2009 13458	Human	Genital	Clinical	1994	United States	KX574889
HSV-2	2009 31735	Human	Genital	Clinical	1993	United States	KX574890
HSV-2	2009 33778	Human	Genital	Clinical	2009	United States	KX574891
HSV-2	2010 21	Human	Genital	Clinical	2009	United States	KX574895
HSV-2	2010 6416	Human	Genital	Clinical	2010	United States	KX574896
HSV-2	2011 21400	Human	Genital	Clinical	1995	United States	KX574908
HSV-2	2011 21761	Human	Genital	Clinical	1998	United States	KX574897
HSV-2	2011 33490	Human	Genital	Clinical	2011	United States	KX574898
HSV-2	2012 32825	Human	Genital	Clinical	2012	United States	KX574901
HSV-2	89 390	Human	Presumed Genital	Clinical *	1989	United States	KR135321
HSV-2	7444 1996 25809	Human	Presumed Genital	Clinical *	1996	United States	KR135314
HSV-2	8937 1999 3336	Human	Presumed Genital	Clinical *	2003	United States	KR135298
HSV-2	10883 2001 13347	Human	Presumed Genital	Clinical *	2005	United States	KR135311
HSV-2	44 319857	Human	Presumed Genital	Clinical *	2007	United States	KR135310
HSV-2	44 419851	Human	Presumed Genital	Clinical *	2007	United States	KR135309
HSV-2	44 619833	Human	Presumed Genital	Clinical *	2007	United States	KR135308
HSV-2	9335 2005 576	Human	Presumed Genital	Clinical *	2009	United States	KR135312
HSV-2	9335 2007 14	Human	Presumed Genital	Clinical *	2011	United States	KR135313
HSV-2	BethesdaP5	Human	Presumed Genital	Clinical **	Unknown	United States	KR135330
HSV-2	GSC 56	Human	Presumed Genital	Low-passage-number clinical	Unknown	United States	KP334094
HSV-2	333	Human	Genital	High-passage-number laboratory	1971	United States	KP192856
HSV-2	COH 3818	Human	Presumed Genital	Low-passage-number clinical	Unknown	United States	KP334096
HSV-2	CtSF	Human	Presumed Genital	Low-passage-number clinical	Unknown	United States	KP334093
HSV-2	1192	Human	Genital	Low-passage-number clinical	Unknown	United States	KP334095
HSV-2	HG52	Human	Anal	High-passage-number laboratory	Prior to 1971	Scotland, UK	JN561323
HSV-2	HSV2 H1421	Human	-	-	-	Finland	KY922723
HSV-2	HSV2 H12212	Human	-	-	-	Finland	KY922726
HSV-2	HSV2 H1227	Human	-	-	-	Finland	KY922721
HSV-2	HSV2 H1526	Human	-	-	-	Finland	KY922724
HSV-2	HSV2 H1226	Human	-	-	-	Finland	KY922720
HSV-2	HSV2 H1229	Human	-	-	-	Finland	KY922722
HSV-2	2009 4550	Human	-	-	2008	Kenya	KX574893
HSV-2	2009 4556	Human	-	-	2008	Kenya	KX574894
HSV-2	N1	Human	Genital	Clinical	Prior to 2007	Norway	EU106422
HSV-2	N2	Human	Genital	Clinical	Prior to 2007	Norway	EU106423
HSV-2	N3	Human	Genital	Clinical	Prior to 2007	Norway	EU106424
HSV-2	N5	Human	Genital	Clinical	Prior to 2007	Norway	EU106426
HSV-2	N6	Human	Genital	Clinical	Prior to 2007	Norway	EU106427
HSV-2	N7	Human	Genital	Clinical	Prior to 2007	Norway	EU106428
HSV-2	N8	Human	Genital	Clinical	Prior to 2007	Norway	EU106429
HSV-2	N9	Human	Genital	Clinical	Prior to 2007	Norway	EU106430
HSV-2	N10	Human	Genital	Clinical	Prior to 2007	Norway	EU106431
HSV-2	2004 4373	Human	-	-	2003	Peru	KX574861
HSV-2	2006 30937	Human	-	-	2005	Peru	KX574866
HSV-2	2006 30839	Human	-	-	2005	Peru	KX574865
HSV-2	2006 45028	Human	-	-	2005	Peru	KX574867
HSV-2	2007 38205	Human	-	-	2004	Peru	KX574860
HSV-2	2007 38249	Human	-	-	2004	Peru	KX574872
HSV-2	2007 38606	Human	-	-	2004	Peru	KX574873
HSV-2	2007 38627	Human	-	-	2004	Peru	KX574874
HSV-2	2007 38807	Human	-	-	2005	Peru	KX574875
HSV-2	2007 38901	Human	-	-	2007	Peru	KX574876
HSV-2	2007 38910	Human	-	-	2006	Peru	KX574877
HSV-2	2007 39303	Human	-	-	2007	Peru	KX574878
HSV-2	2007 39521	Human	-	-	2005	South Africa	KX574879
HSV-2	SD90e	Human	Genital	Low-passage-number clinical	1994	South Africa	KF781518
HSV-2	2007 39712	Human	-	-	2006	South Africa	KX574880
HSV-2	2007 39729	Human	-	-	2007	South Africa	KX574881
HSV-2	2009 3532	Human	-	-	2007	South Africa	KX574892
HSV-2	S 93 4530	Human	Genital	Clinical	Prior to 2007	Sweden	EU106468
HSV-2	S 97 1643	Human	Genital	Clinical	Prior to 2007	Sweden	EU106466
HSV-2	S B4327	Human	Genital	Clinical	Prior to 2007	Sweden	EU106469
HSV-2	2009 409	Human	-	-	2006	Tanzania	KX574902
HSV-2	T 1855	Human	Genital	Clinical	Prior to 2007	Tanzania	EU106440
HSV-2	T 2032	Human	Genital	Clinical	Prior to 2007	Tanzania	EU106439
HSV-2	T 2302	Human	Genital	Clinical	Prior to 2007	Tanzania	EU106432
HSV-2	T 2557	Human	Genital	Clinical	Prior to 2007	Tanzania	EU106436
HSV-2	T 2737	Human	Genital	Clinical	Prior to 2007	Tanzania	EU106438
HSV-2	T 3034	Human	Genital	Clinical	Prior to 2007	Tanzania	EU106433
HSV-2	T 3041	Human	Genital	Clinical	Prior to 2007	Tanzania	EU106441
HSV-2	T 3111	Human	Genital	Clinical	Prior to 2007	Tanzania	EU106434
HSV-2	T 4587	Human	Genital	Clinical	Prior to 2007	Tanzania	EU106435
HSV-2	T 2228 01	Human	Genital	Clinical	Prior to 2007	Tanzania	EU106443
HSV-2	T 2316 10	Human	Genital	Clinical	Prior to 2007	Tanzania	EU106444
HSV-2	T 2346 14	Human	Genital	Clinical	Prior to 2007	Tanzania	EU106445
HSV-2	T 28 2595	Human	Genital	Clinical	Prior to 2007	Tanzania	EU106447
HSV-2	T 35 2769	Human	Genital	Clinical	Prior to 2007	Tanzania	EU106448
HSV-2	T 43 742	Human	Genital	Clinical	Prior to 2007	Tanzania	EU106450
HSV-2	T 47 2929	Human	Genital	Clinical	Prior to 2007	Tanzania	EU106452
HSV-2	T 53 1390	Human	Genital	Clinical	Prior to 2007	Tanzania	EU106454
HSV-2	T 56 3127	Human	Genital	Clinical	Prior to 2007	Tanzania	EU106455
HSV-2	T 70 3486	Human	Genital	Clinical	Prior to 2007	Tanzania	EU106456
HSV-2	T NN1	Human	Genital	Clinical	Prior to 2007	Tanzania	EU106458
HSV-2	2011 34727	Human	-	-	2011	Uganda	KX574899
HSV-2	2012 9333	Human	-	-	2011	Uganda	KX574906
HSV-2	2006 19390	Human	-	-	2005	Zambia	KX574862
HSV-2	2006 29060	Human	-	-	2005	Zambia	KX574864
HSV-2	2007 8222	Human	-	-	2006	Zambia	KX574883
HSV-2	2009 2198	Human	-	-	2006	Zambia	KX574905
HSV-2	3006 21832	Human	-	-	2005	Zimbabwe	KX574863
HSV-2	2007 38120	Human	-	-	2004	Zimbabwe	KX574871
ChHV-1	105640	Chimpanzee	Oral	Clinical	2004	United States	NC023677

Underlined GenBank accession numbers were sequenced in this research.

- No information was provided in the source document.

* Isolates underwent two passages on Vero cells.

** This isolate underwent four passages on human diploid fibroblasts.

### Non-synonymous (*d*N), and synonymous (*d*S) substitutions

The frequencies of non-synonymous (*d*N), and synonymous (*d*S) substitutions and *d*N/*d*S ratios for each of the *U*_*S*_*7*, *U*_*S*_*8*, *U*_*S*_*8A*, and *U*_*S*_*9* genes were calculated using the SNAP (Synonymous Non-synonymous Analysis Program) v2.1.1 website [27]. A *d*N/*d*S ratio of less than one indicates negative purifying selection, meaning that natural selection suppresses protein changes. A *d*N/*d*S ratio of greater than one indicates positive selection. A *d*N/*d*S ratio equal to one indicates selection neutrality where non-synonymous substitutions occur at a rate equal to synonymous substitutions [28]. The difference in mean ratios of non-synonymous (*d*N) to synonymous (*d*S) evolutionary substitutions in HSV-2 *U*_*S*_*7*, *U*_*S*_*8*, *U*_*S*_*8A*, and *U*_*S*_*9* from subjects with non-recurrent vs. recurrent disease isolates were then compared for significance by two-tailed t-tests (p < 0.05).

### Phylogenetic analyses

Two HSV-2 phylogenies were used to evaluate the evolutionary relationships between HSV-2 strains using the concatenated sequence of the *U*_*S*_*7-U*_*S*_*8-U*_*S*_*8A-U*_*S*_*9* region. Because the main objective of this research is to evaluate whether sequence differences in the gE/gI and US9 proteins obtained from HSV-2 U.S. isolates are associated with more frequent recurrence, a U.S.-restricted phylogeny was first calculated. This phylogeny encompassed 55 HSV-2 isolates from the U.S. (including the 12 non-recurrent and 8 recurrent isolates sequenced from the Herpevac Trial). Sequences were aligned to the sequence of the South African clinical isolate SD90e, and the ChHV-1 sequence was included as an outgroup (Table 2). Second, a phylogeny was calculated using HSV-2 *Us7-Us9* sequences from different parts of the world (Table 2): 55 sequences from the U.S., 12 from Peru, 19 from Northern Europe, 5 South African, 21 Tanzanian, and 10 from other SoutheastAfrican countries. Bayesian Inference (BI), Maximum parsimony (MP) and Maximum Likelihood (ML) based searches (including coding and non-coding positions) were performed for both the independent and the combined data sets using, respectively, *MrBayes 3*.*2*.*6* [29, 30], PAUP* *4*.*0 beta10* [31], and *PhyML 3*.*0* [32]. All gaps were treated as missing data. Tests of goodness of fit for alternative nucleotide substitution models were performed in *jModelTest* 2 using the Akaike information criterion (AIC) [33], Bayesian Information Criterion (BIC) [34] and a decision-theoretic (DT) performance-based approach [35–37]. *jModelTest* 2 selected TPM1uf+I as the optimal model. The closest GTR+G model was imposed in the respective partitions for the BI and ML inferences. BI analyses were run with one million generations using the Monte Carlo Markov Chain (MCMC) algorithm. Trees were sampled every 1,000 generations, and 25% of the generations were discarded as burn-in once stability in the likelihood values was attained. A half-compatible consensus Bayesian tree was computed from the 750 posterior probability saved trees. ML analysis was computed using 20 starting trees from 20 distinct randomized MP trees and 1000 bootstrap (BS) replicates for analysis of U.S. strains. MP analysis was based on heuristic searches of 10,000 random-order-entry trees, with TBR branch swapping and saving no more than 10 trees per replicate. The most parsimonious trees were used to compute the respective strict consensus trees. Branch support was estimated through 1,000 bootstrap replicates [38] using the TBR-M (Tree Bisection Reconstruction swapping, MULPARS OFF) strategy of DeBry [39] as a method to reduce computational time. Clusters were considered strongly supported when at least two out of the three supporting methods tested indicated elevated support: Bayesian Posterior Probabilities (≥80%); Maximum-Parsimony Bootstrap (≥65%); and Maximum-Likelihood Bootstrap (≥65%).

The phylogenetic analyses were also used to identify *U*_*S*_*7*, *U*_*S*_*8*, *U*_*S*_*8A*, and *U*_*S*_*9* SNVs shared within and between different HSV-2 U.S. clusters. SNVs were identified by ordering the nucleotide (3,768 genic and intergenic nucleotide positions) and amino acid sequences [US7 (gI), 1131 nucleotides/377 amino acids; US8 (gE), 1647/549; US8A, 441/147; US9, 270/90] of each strain sampled according to the phylogenetic tree and conducting a detailed visual examination of the concatenated sequences.

## Results

### Sample selection and vaccination

Twelve subjects were identified who became infected with HSV-2 and had a single disease episode during the Herpevac Trial; here we define isolates from these subjects as “non-recurrent.” Eight subjects had 3 to 7 recurrences during the Trial. We sequenced isolates from the last disease episode of these subjects who had 3 or more recurrences during the trial and define these as “recurrent.” Among the subjects with multiple recurrent disease episodes, the 4 subjects who received the control hepatitis A vaccine (HAV) had an average of 4.75 recurrent episodes of disease, while the 4 who received the investigational HSV vaccine had an average of 3.75 recurrent episodes of disease (p > 0.05). The average number of days between recurrent disease episodes was 38.8 for HAV vaccine recipients, compared with an average of 54.4 days for those who received the HSV vaccine (p > 0.05).

### Sequence analyses

**Fig 1:**
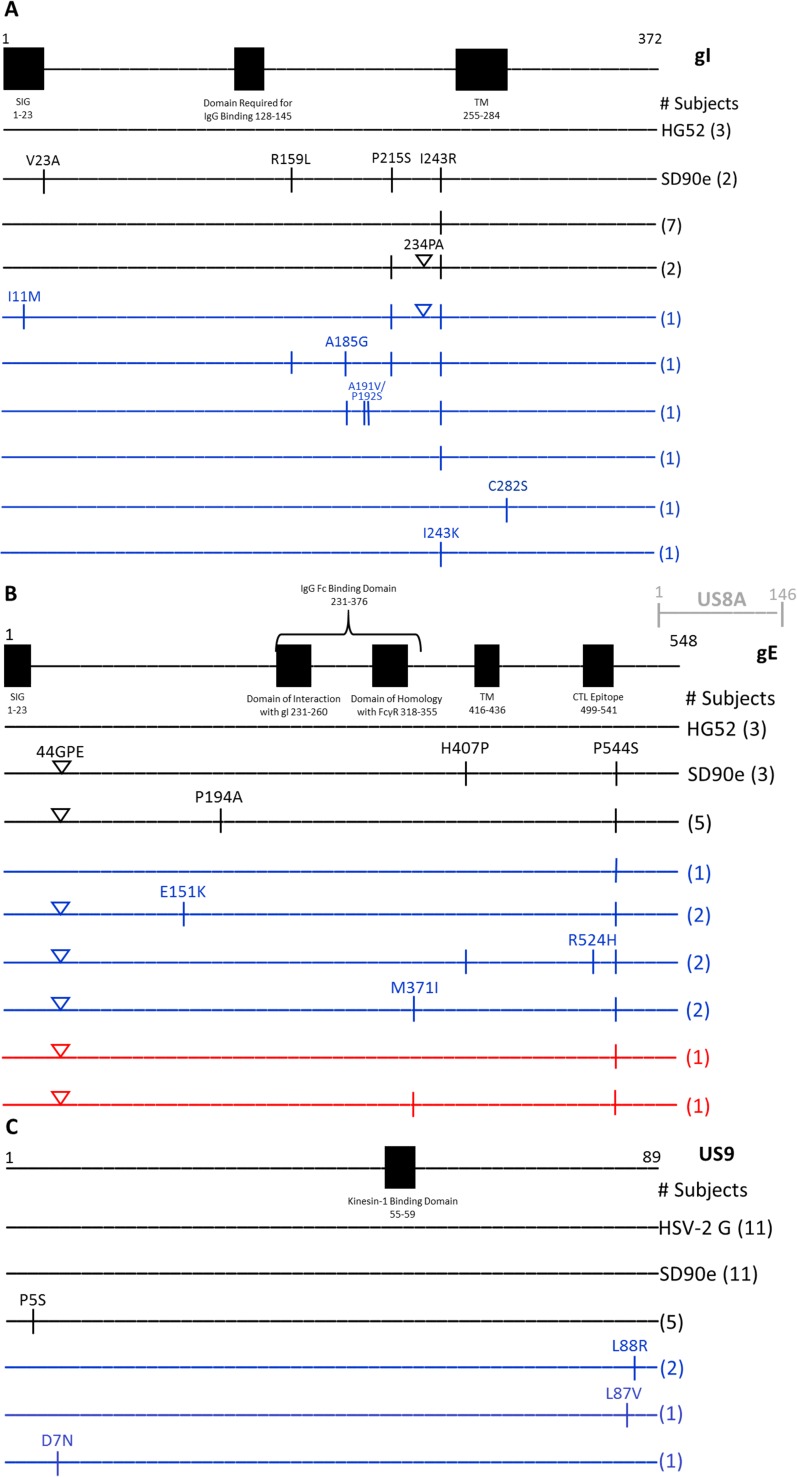
gI, gE and US9 amino acid sequences of isolates from the Herpevac Trial. Substitutions in the A) gI, B) gE, and C) US9 amino acid sequences are shown compared to reference sequence HG52. Sequence of the South African reference strain SD90e is also represented. Colored lines indicate whether the pattern of substitutions occurred in non-recurrent and recurrent samples (black), non-recurrent samples only (blue) or recurrent samples only (red). Vertical lines represent substitutions and triangles represent INDELs. The uppermost example of each variant is labeled; those below share the same variation unless indicated otherwise (e.g. I241K vs. I241R). The number of subjects’ samples that match each pattern is indicated at the right in parentheses. The location of the putative US8A polypeptide is also shown. SIG, signal sequence; tm, transmembrane domain.

First, polymorphisms in *Us7-Us9* among non-recurrent isolates and recurrent isolates were identified by comparison of the nucleotide sequences to the HSV-2 HG52 strain as reference. Aligned sequences were translated and isolates that had the same pattern of amino acid polymorphisms were grouped together. These groups were then classified as consisting of non-recurrent isolates only, recurrent isolates only, or both non-recurrent and recurrent isolates (Fig 1A–1C). In gI, both non-recurrent and recurrent isolates contained V23A, R159L, P215S, 234PA, and I243R. An additional 232PAPA was found in one recurrent isolate. Polymorphisms I11M, A185G, A191V, P192S, I243K, and C282S were unique to gI of non-recurrent isolates, although I11M and V23A fell within the proposed signal peptide (15). No gI polymorphisms were unique to recurrent isolates (Fig 1A). In gE, polymorphisms 44GPE, P194A, M371I, H407P, and P544S occurred in both non-recurrent and recurrent isolates, while E151K, and R524H were found only in non-recurrent isolates. No gE polymorphisms were unique to recurrent isolates (Fig 1B). The HSV-2 *Us7-Us9* complex contains another open reading frame homologous to *Us8A* of HSV-1. The only identified polymorphism within the translated *Us8A* open reading frame, A26T, was found in non-recurrent (subject 2) and recurrent (subject 32) isolates. In US9, polymorphisms P5S was found in both non-recurrent and recurrent isolates while D7N, L87V and L88R were found only in non-recurrent isolates. No US9 polymorphisms were unique to recurrent isolates (Fig 1C).

### Non-synonymous (*d*N), and synonymous (*d*S) substitutions

**Fig 2:**
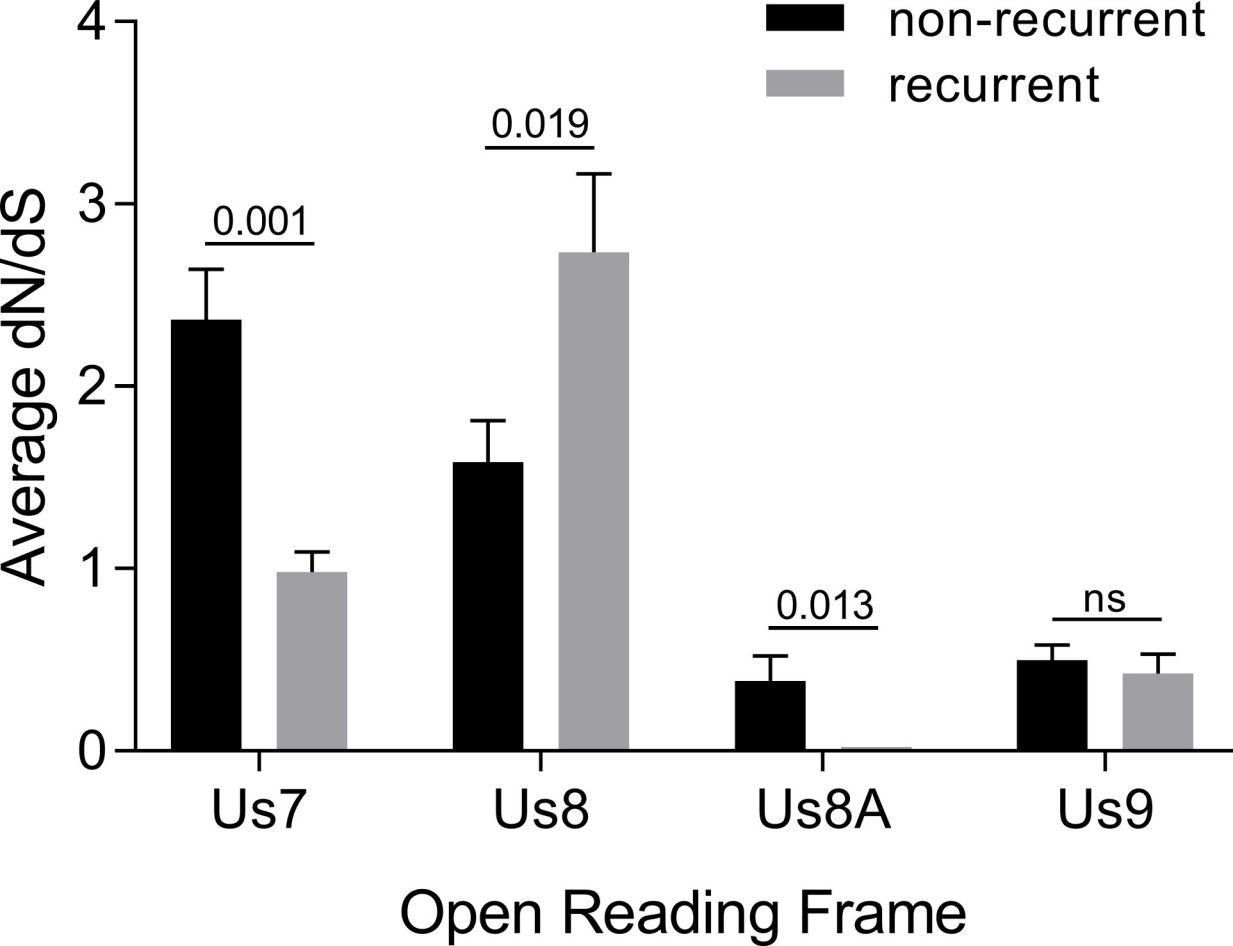
Mean ratios of non-synonymous (*d*N) to synonymous (*d*S) evolutionary substitutions in Us7, Us8, Us8A, and Us9 genes from subjects with non-recurrent vs. recurrent HSV-2 infections. Bars represent the mean ± SEM of 12 non-recurrent or 8 recurrent samples. P values are shown above the bars. ns, not statistically significant.

The frequencies of non-synonymous (*d*N), and synonymous (*d*S) substitutions were calculated based on the codon-aligned nucleotide sequences in a data set that included the 12 non-recurrent and 8 recurrent isolates, and then *d*N/*d*S ratios were determined (Fig 2). The *d*N/*d*S ratio for *Us7* was significantly higher in non-recurrent isolates compared to frequently recurring subjects’ isolates, with average *d*N/*d*S ratios of 2.37 and 0.98 respectively (p = 0.001). Like *Us7*, *Us8A* had a significantly higher *d*N/*d*S ratio in non-recurrent isolates than recurrent isolates, with an average *d*N/*d*S ratio of 0.38 for non-recurrent isolates and 0.02 for recurrent isolates (p = 0.013), although *Us8A* ratios for both sets of isolates were substantially <1. The *d*N/*d*S ratios for *Us9* of non-recurrent and recurrent isolates were also both substantially <1 but not significantly different. Conversely, the *d*N/*d*S ratio of *Us8* was significantly lower in non-recurrent isolates than frequently recurring subjects’ isolates, with average *d*N/*d*S ratios of 1.59 and 2.74 respectively (p = 0.019). Notably, both *d*N/*d*S ratios for *Us8* were >1.

### Phylogenetic reconstruction and nucleotide variations shared between non-related strains

**Fig 3:**
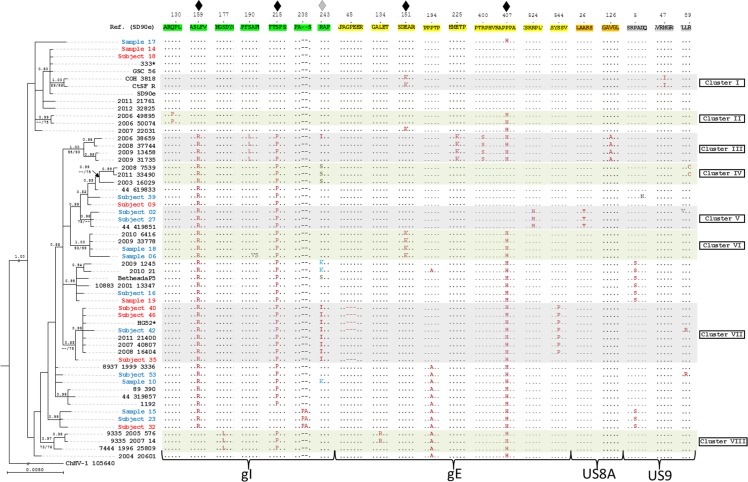
Phylogenetic reconstruction and amino acid variants shared among different U.S. HSV-2 clusters. Amino acid sequences in the SD90e reference strain correspond to gI (highlighted in green), gE (yellow), US8A (orange) and US9 (grey). Each gene segment is also bracketed at the bottom of the figure. Amino acid sequences were ordered according to the Bayesian tree diagrammed on the left side of the figure. Blue and red accession numbers represent, respectively, non-recurrent and recurrent isolates (Table 2). Selected regions with amino acid variation are shown. The position of each amino acid variant shared by non-related clusters (♦) or at which multiple amino acid variants occur (♦) is indicated above the reference strain. Bayesian posterior probabilities (≥80%) and maximum-parsimony bootstrap (≥65%) / maximum-likelihood bootstrap (≥65%) values appear above and below the branches, respectively. * High-passage laboratory strain.

The 20 HSV-2 sequences obtained in this study provided an additional dataset for assessing the phylogenetic patterns among U.S. HSV-2 strains. The concatenated *U*_*S*_*7*, *U*_*S*_*8*, *U*_*S*_*8A*, and *U*_*S*_*9* sequences from a total of 55 strains plus the HG52 and 333 laboratory strains (Table 2) were aligned to SD90e and analyzed using ChHV-1 as an outgroup. The phylogenetic reconstruction revealed 8 well-supported clusters (Fig 3 and S1 Fig). Detailed examination of the sequences revealed six nucleotide variations shared between non-related strains (S1 Fig), four of which were non-synonymous and two synonymous (S1 Fig). Interestingly, non-synonymous nucleotide variations in positions 728 and 729 of *U*_*S*_*7* generated three different amino acids in position 243 (isoleucine, serine and lysine; Fig 3). No crystal structure models of homologs for these HSV proteins exist so the biological significance of the polymorphisms, if any, is unclear. Next, the 55 U.S. strains were incorporated into a broader phylogenetic analysis of HSV-2 *U*_*S*_*7-U*_*S*_*9* sequences from a total of 121 strains from different parts of the world. The global phylogenetic reconstruction (including strains from South America, North Europe, and Southeast Africa; Table 2 and S2 Fig) revealed the same eight strongly supported clusters, several of which had expanded to include more isolates. In addition, five new small but strongly supported clusters appeared.

## Discussion

A powerful aspect of this study is that participants in the Herpevac Trial for Women were HSV-1/2 seronegative at outset and had periodic serum samples drawn so their first episode of disease is well documented. Thus, “non-recurrent” individuals could be clearly defined and distinguished from individuals having multiple disease episodes. Our analysis of 20 HSV-2 isolates from the Herpevac Trial did not reveal a nucleotide or amino acid variant that tracked with frequent recurrence. However, differences in the *d*N/*d*S ratios of non-recurrent and recurrent isolates were found, suggesting that certain selection pressures act on each protein. The average *d*N/*d*S ratio for *Us7* (gI) and *Us9* were significantly higher in non-recurring isolates than the average for the frequently recurring isolates. These results suggest gI and US9 amino acid sequences trend toward a consensus sequence in infected individuals who have frequent disease episodes. Conversely, *Us8* (gE)’s *d*N/*d*S ratio was significantly higher in recurrent (2.74) than non-recurrent (1.59) isolates, and both were >1, suggesting that variation in gE confers an advantage to the virus, particularly over time in infected persons. Our sample size was relatively small; possibly a larger dataset would reveal a consistent variation that differentiates frequently recurring viruses. The average *d*N/*d*S ratios of *Us8A* from non-recurrent and recurrent isolates were both close to zero, supporting a proposed role of its putative gene product, by analogy to HSV-1 US8A, in invasion of the CNS from peripheral sites [18]. The capacity to reach the nervous system to establish latency is a critical part of the virus’ life cycle. Thus, HSV-2 US8A deserves further study.

### Role of sequence variation in virus recurrence

If SNVs accumulate with repeated reactivations of HSV-2 from a latent state, sequences from recurrent strains should contain nucleotide variations not present in non-recurrent sequences. This phenomenon would result in sequence convergence, and recurrent sequences might also appear grouped in phylogenetic trees. We did not observe this pattern in the samples and genes studied.

Variation, however, was not uniform across the open reading frames studied. Importantly, we observed that functional domains in gE/gI and US9 are more conserved than other regions of the sequences (Fig 1). gI and gE must form a heterodimer to facilitate anterograde transport of viral glycoproteins and capsids to axon termini during a reactivation [40]. We found no amino acid changes in gI’s proposed domain of interaction with gE (amino acids 129–145) or gE’s proposed domain of interaction with gI (amino acids 231–260), supporting previous findings that the formation of this heterodimer is an essential event. Axonal transport of capsids and glycoproteins is known to be impaired in US9 null mutants [40], and in fact, no amino acid changes occurred in HSV-2 US9 sequences with homology to the proposed kinesin-1 binding domain of HSV-1 US9 (amino acids 55–59) [41]. These findings support vital roles for gI, gE, and US9 in neuronal transport.

### Immune escape

Comparison of our HSV-2 sequences to the previously described HSV-1 Fc binding domains of gE and gI [19] demonstrated they have similarly located binding domains of identical sequence (Fig 1A and 1B). The absence of polymorphisms in HSV-2 gE’s domain homologous to the mammalian FcRγ (amino acids 318–355) and gI’s domain required for IgG binding (amino acids 128–145) supports previous findings [19], and suggests that the HSV-2 gE/gI as an FcRγ helps maintain viral fitness. T cell recognition of HSV-infected cells is also a critical component of the immune response. A previously identified CTL epitope (restricted by HLA B*5701) occurs in HSV-2 gE at amino acid positions 518–526 [17]. Alteration of the epitope could favor immune escape and thus successful recurrence in HLA B*5701^+^ individuals. Interestingly, subjects 2 and 27, who did not have recurrent disease, showed variation at gE amino acid position 524. We also observed a variant at position 524 in a previously sequenced isolate of unknown recurrence status, isolate 44_419851 (40). Variation in the HSV-2 gE 518–526 region in non-recurrent isolates could be explained if subjects 2 and 27 had acquired a virus with the gE epitope variant, but they lack the HLAB*5701 allele. Under these circumstances the effect of the variant would be neutral. The high *d*N/*d*S ratio findings for *Us8* (gE) of recurrent isolates also supports the contention that portions of gE may serve as targets of immune recognition and variation in this protein may confer an evolutionary advantage. It must be borne in mind, however, that evasion of one CTL epitope does not constitute escape from the whole immune arsenal.

### Phylogenetic reconstruction

Both phylogenetic reconstructions using the 20 study samples and 25 additional U.S. sequences or 101 additional geographically diverse sequences downloaded from GenBank revealed eight well-supported clusters of the concatenated *Us7-Us9* region (Fig 3 and S1 and S2 Figs). In contrast to previous studies that focused on nearly complete HSV-2 genomes [42–44], we assessed the phylogenetic relationships among specifically the *U*_*S*_*7-U*_*S*_*9* glycoprotein region, including coding and non-coding positions. Two salient observations arose: First, we did not detect a clear pattern that demonstrates a phylogenetic association among non-recurrent or recurrent isolates. Thus, in concordance with a preceding hypothesis [42], SNVs that accumulate with repeated reactivations of HSV-2 from a latent state do not necessarily lead to sequence convergence. However, the amount of time virus coexists with the host may influence sequence convergence and the recurrence history of GenBank strains included in our phylogenetic study is unknown. Second, the phylogeny observed for clinical isolates is generally similar to the phylogenetic relationships described for the same clinical samples but using the complete HSV-2 genome [42–44]. For example, some clusters observed in our *Us7-Us9* phylogeny (e.g. cluster I and VIII) have been previously described in other U.S. HSV-2 phylogenies based on the nearly complete genomes [42, 43]. Nevertheless, several exceptions were observed (e.g. sample 14 and 19), probably due to the limited genomic scope of a survey of the *U*_*S*_*7-U*_*S*_*9* complex (3,768 nt) compared with the whole HSV-2 genome (~154,000 nt).

### Nucleotide variations shared between non-related strains

Detailed examination of the nucleotide and amino acid sequences of the glycoprotein *U*_*S*_*7-U*_*S*_*9* complex in 55 U.S. HSV-2 sequences revealed six nucleotide variations shared between strains nested in different clusters (S1 Fig), four of which were non-synonymous (Fig 3). Polymorphisms in coding regions may allow a variant to emerge which could, for example, evade host immune surveillance or adapt to a new host’s genetic makeup. However, lack of homologous crystal structure models precluded interpretation of their significance. At least two evolutionary processes could explain the presence of identical nucleotide variations among non-related strains. The parallel nucleotide substitution theory has been previously invoked to explain analogous evolutionary events in diverse groups [45], and mutation-driven parallel evolution during viral adaptation [46]. However, random mutation leading to homoplasy is improbable based on the high number of identical mutations in non-related taxa observed in the small *U*_*S*_*7-U*_*S*_*9* complex (3,768 nt). Alternatively, homologous recombination has been previously described as part of the evolution of many alphaherpesviruses [47], particularly within the HSV-2 genome [43, 44, 48, 49], and within and between HSV-2 glycoproteins and their HSV-1 counterparts [50]. Thus, the most likely explanation for the six nucleotide variations shared between non-related clusters (Fig 3 and S1 Fig) is recombination. Previous phylogenetic studies of HSV-2 have concluded that determining inter-strain phylogenies is difficult due to extensive recombination, leading to an underestimate of phylogenetic groupings [43, 48]. This recombination is implied by inconsistent phylogenetic signals across sequence alignments in Bootscan analysis, which suggests that HSV-2 strains observed are a genetic mosaic [43].

“Non-recurrent” isolates were defined as samples from subjects who became infected but did not have recurrent disease within the 20-month observation period post vaccine dose 2. Though these subjects have an apparently less severe disease course because first disease recurrence is likely to occur within the first year [51], they may not be strictly “non-recurrent” if they had been followed over a longer time period or if they had had subclinical reactivations. Only 8 individuals in this study who became infected with HSV-2 during the Trial had recurrent episodes of disease, so our sample size for analyzing sequence variations associated with recurrence is small. Additional vaccine trials may expand our dataset and allow a variant associated with recurrence to emerge.

## Supporting information

S1 FigPhylogenetic reconstruction and nucleotide variants shared among different U.S. HSV-2 clusters.Nucleotide sequences in the SD90e reference strain correspond to *Us7* (highlighted in green), *Us8* (yellow), *Us8A* (orange) and *Us9* (grey). In addition to the color coding, each gene segment is also labeled with a bracket in the bottom of the figure. Nucleotide sequences were ordered according to the Bayesian tree diagrammed on the left side of the figure. Blue and red accession numbers represent, respectively, non-recurrent and recurrent isolates (Table 2). A) Regions with variation between nucleotides 244 and 1910; B) regions with variation between nucleotides 1995 and 3759. Colored bands represent clusters supported by two different methods of analysis. The position of each nucleotide variation shared by non-related clusters is indicated above the reference strain (♦, non-synonymous and ♢, synonymous). The nucleotide positions at which multiple SNVs occur (♦) is also indicated. Bayesian posterior probabilities (≥80%) and maximum-parsimony bootstrap (≥65%) / maximum-likelihood bootstrap (≥65%) values appear above and below the branches, respectively. * High-passage laboratory strain.(TIF)

S2 Fig Bayesian Majority Rule consensus tree of HSV-2 based on the *Us7-Us9* region (coding and non-coding sequences). The phylogeny of HSV-2 strains collected from diverse parts of the world (see color key and Table 2) with special emphasis on strains sampled in the U.S. (bold black font). Blue and red entries represent, respectively, U.S. non-recurrent and recurrent HSV-2 isolates sequenced in this research. Bayesian posterior probabilities (≥80%) appear above the branches and Maximum-Parsimony bootstrap (≥65%) / Maximum-Likelihood bootstrap (≥65%) values appear below the branches. The eight different clusters from Fig 3 and S1 Fig are noted on the right side of the tree. A) Upper half of the phylogenetic tree; B) lower half of the tree.
(TIF)
