# Supplementary figures and images for: Molecular analyses and phylogeny of the herpes simplex virus 2 US9 and glycoproteins gE/gI obtained from infected subjects during the Herpevac Trial for Women
Source: PLoS One. 2019 Mar 8;14(3):e0212877. doi: 10.1371/journal.pone.0212877 (PMC6407778; doi:10.1371/journal.pone.0212877)

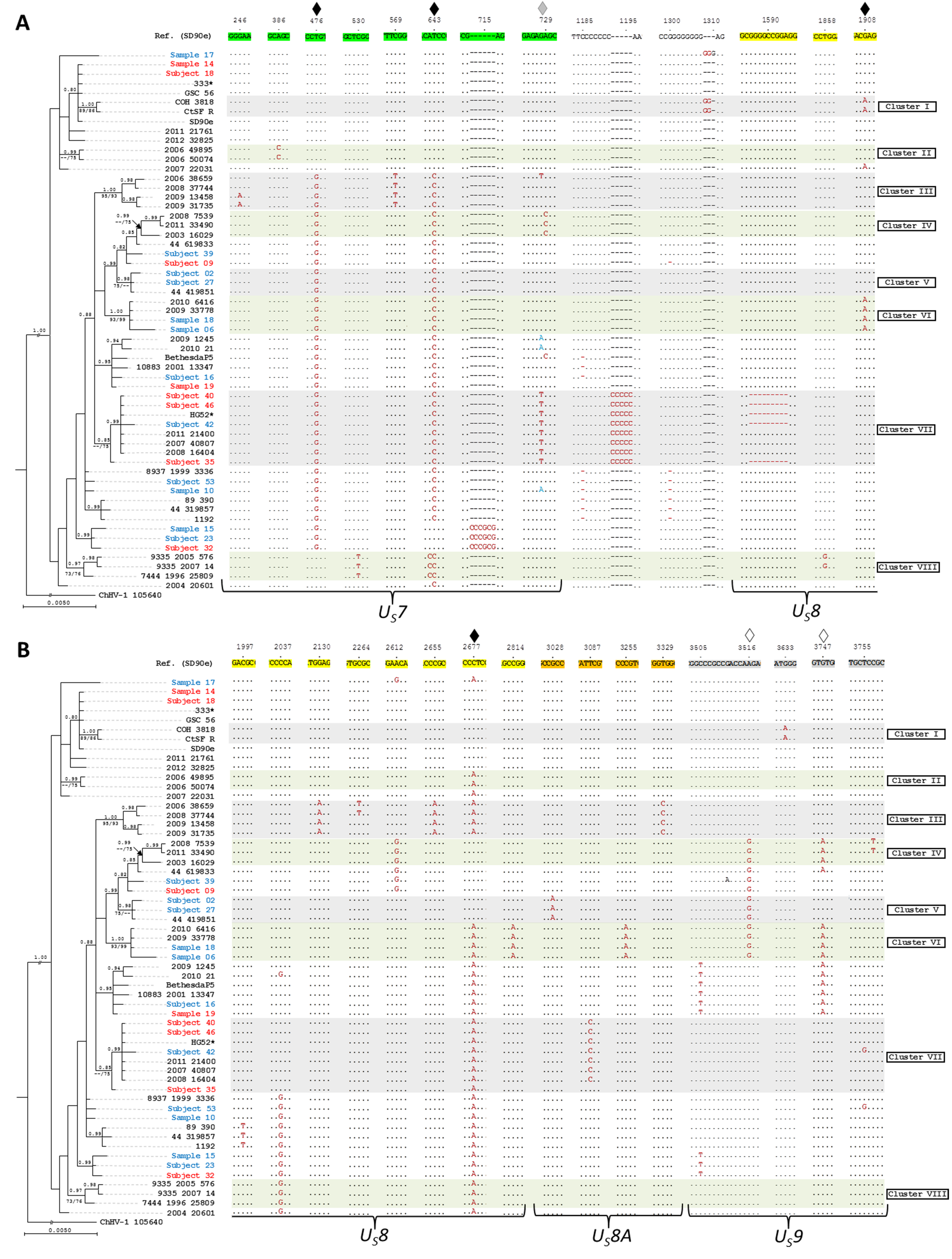

Supplement: S1 Fig — Nucleotide sequences in the SD90e reference strain correspond to Us7 (highlighted in green), Us8 (yellow), Us8A (orange) and Us9 (grey). In addition to the color coding, each gene segment is also labeled with a bracket in the bottom of the figure. Nucleotide sequences were ordered according to the Bayesian tree diagrammed on the left side of the figure. Blue and red accession numbers represent, respectively, non-recurrent and recurrent isolates (Table 2). A) Regions with variation between nucleotides 244 and 1910; B) regions with variation between nucleotides 1995 and 3759. Colored bands represent clusters supported by two different methods of analysis. The position of each nucleotide variation shared by non-related clusters is indicated above the reference strain (♦, non-synonymous and ♢, synonymous). The nucleotide positions at which multiple SNVs occur (♦) is also indicated. Bayesian posterior probabilities (≥80%) and maximum-parsimony bootstrap (≥65%) / maximum-likelihood bootstrap (≥65%) values appear above and below the branches, respectively. * High-passage laboratory strain. (TIF) [file pone.0212877.s001.tif]

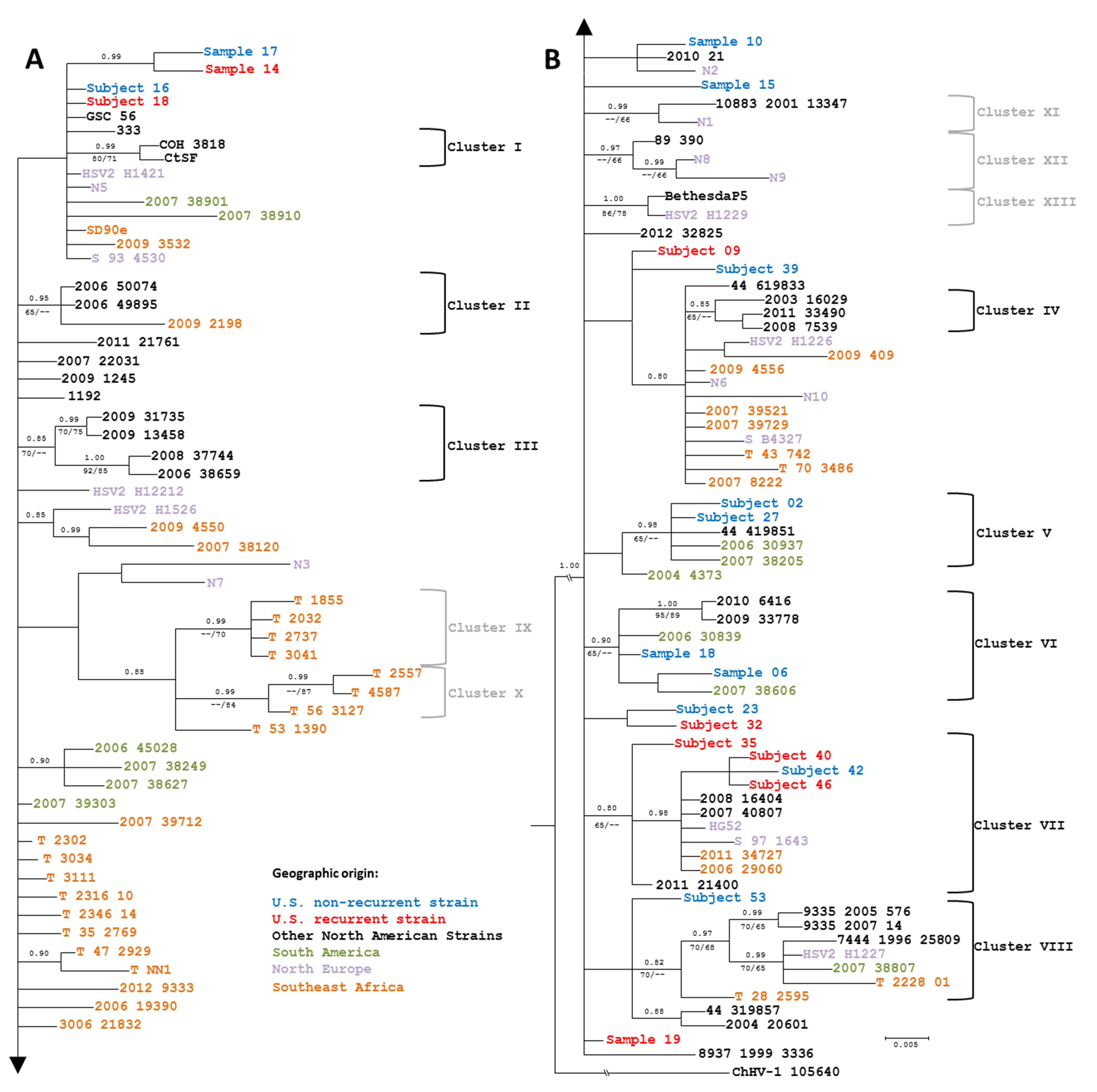

Supplement: S2 Fig — The phylogeny of HSV-2 strains collected from diverse parts of the world (see color key and Table 2) with special emphasis on strains sampled in the U.S. (bold black font). Blue and red entries represent, respectively, U.S. non-recurrent and recurrent HSV-2 isolates sequenced in this research. Bayesian posterior probabilities (≥80%) appear above the branches and Maximum-Parsimony bootstrap (≥65%) / Maximum-Likelihood bootstrap (≥65%) values appear below the branches. The eight different clusters from Fig 3 and S1 Fig are noted on the right side of the tree. A) Upper half of the phylogenetic tree; B) lower half of the tree. (TIF) [file pone.0212877.s002.tif]
